# Destigmatizing alcohol use disorder among nurses

**DOI:** 10.1097/01.NURSE.0000832364.28141.12

**Published:** 2022-06-23

**Authors:** Jill Rathburn

**Affiliations:** **Jill Rathburn** is a board-certified recovery coach and holistic health nurse.

**Keywords:** addiction, alcohol abuse, alcohol dependence, alcohol use disorder, alternative-to-discipline program, DSM-5, nursing, stigma

## Abstract

The use of alcohol to cope with work-related stress is an increasing problem among nurses. However, barriers to diagnosis and treatment keep nurses with alcohol use disorder (AUD) from getting the help they need. This article discusses the issues and treatment obstacles affecting AUD among nurses, and outlines compassionate, stigma-free paths forward.

## Abstract 2

The use of alcohol to cope with work-related stress is an increasing problem among nurses. This article discusses the issues and treatment obstacles affecting alcohol use disorder among nurses, and outlines compassionate, stigma-free paths forward.

**Figure:**
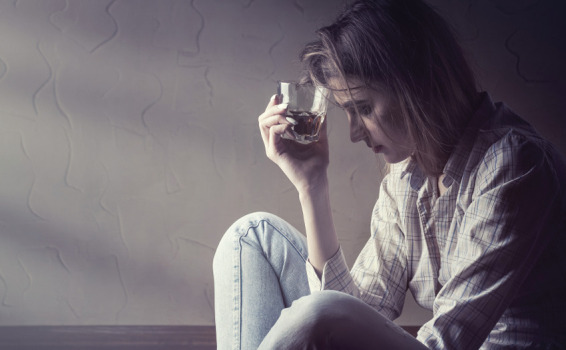
No caption available.

Sarah was the nurse version of Superwoman. She started full-time on the night shift, quickly rose to preceptor, and was promoted to charge nurse. She had two children, and she later took on a second job to supplement her income—all the while building a loving marriage and family life. She found that alcohol was the quickest way to come down from long, dizzying days and quiet her mind for a few hours of sleep. First, she had a little alcohol, then a little more, then a lot more. She started developing an alcohol use disorder (AUD).

Due to a work injury, Sarah later underwent two surgeries and was told that she would no longer be able to provide bedside clinical care. Sarah's alcohol use progressed, and her problems multiplied: a Driving Under the Influence (DUI) offense, an attempt at sobriety, then another DUI offense.

During her second rehabilitation admission, the treatment facility contacted the state board of nursing (BON), even though she was not currently working and had never worked intoxicated or hungover. Sarah's employment record was exemplary, but the BON asked her to voluntarily surrender her nursing license until she could prove a year of sobriety.

After Sarah completed her first year of sobriety, she expected to receive direction from the BON on how to reinstate her nursing license. Instead, she was met with resistance. Her frustration, along with other stressors, contributed to periodic relapses. Nonetheless, as she regained her year-long sobriety, she would periodically contact the BON to ask for guidance only to hear, “read your stipulation” without further direction.

She was later introduced to her state's Nurses Peer Support Network, where she found her roadmap back into the profession as a nursing assistant. Here, she rediscovered her roots in healthcare with the support of her supervisor. However, she was taken aback by her colleagues' habitual after-work drinking while casting judgment on anyone who “had a drinking problem.”

Unfortunately, Sarah's story is not uncommon. It is important to address why this type of scenario repeatedly unfolds among nurses with AUD.

## Hiding in plain sight

Over 20 nurses from a national recovery support group volunteered to be interviewed for this article (see *Interview questions on the history of AUD and workplace support*). Without exception, they believed that alcohol abuse was unquestionably on the rise among nurses, particularly since the start of the COVID-19 pandemic.

They noted their nursing colleagues had been talking a lot more about drinking and increasing their after-hours drinking to cope with stress. Allegedly, one supervisor unsarcastically suggested her staff drink a bottle of wine to take the edge off.

The interviewed nurses also reflected on “mommy wine culture,” where parents socially consume wine as a coping mechanism for the everyday stressors of parenting.^1^ “Alcohol is everywhere,” one nurse shared. “No one sees the dangers, and the packaging is cute and innocent as a sippy cup.”

Alcohol consumption has become so normalized in the US that its place on the slippery slope of addiction seems much harder to recognize.^2^ Two cocktail party drinks or that nightcap after a long day in the clinic often grows into many more.

In a recent study on substance use among nurses in Indiana, survey participants were asked to comment on substance or alcohol use they had experienced or witnessed among RNs.^3^ The researchers found repeated evidence showing that the use of alcohol to cope with professional stress may be an increasing problem in nursing.^3^

More data emerged with the 2022 Nurse Worklife and Wellness Study, which sought to address the incidence of substance use disorder (SUD) among nurses. This study evaluated the use of numerous substances, including alcohol, among 1,170 nurses. A total of 354 nurses (31% of the survey participants) reported drinking alcohol within the past year. Of those, 124 nurses (35%) who consumed alcohol only screened positive for substance use problems or SUD, and 231 nurses (65.4%) who consumed both alcohol and drugs screened positive for substance use problems or SUD.^4^

While both studies suggest a growing problems with alcohol use among nurses, the researchers used instruments for assessing SUD, not AUD. To best understand the prevalence of AUD, it is essential to use the *Diagnostic and Statistical Manual of Mental Disorders* (DSM-5) diagnostic criteria for AUD.^5^

These studies signal that alcohol use is an increasing problem among nurses. This article discusses the issues and treatment obstacles affecting AUD among nurses, and outlines compassionate, stigma-free paths forward.

## Comprehensive AUD data needed

Notably, the study of AUD is absent from the medical literature. Studies designed to capture AUD-specific data among nurses are needed to address the complete scope of this problem.

For example, in a recent systematic review of more than 30 studies that examined the psychological health impacts of COVID-19 on healthcare professionals (HCPs), 8 outcomes including anxiety and SUD were addressed. However, issues related to alcohol or AUD were not mentioned.^6^

Because there is a dearth of literature focusing solely on AUD prevalence among nurses, and given that 91% of US nurses are women, it can be useful to consider the current AUD data on the broader population of women in the US.^7^ Women have increased their heavy drinking days (four or more drinks within a couple of hours/day) by 41% compared with prepandemic levels based on a recent study published in *JAMA*.^8^ Researchers also reported the psychological stress related to COVID-19 was associated with increased drinking for women and found a 39% increase in women's “alcohol-related problems” since 2019.^8^

The impediments to a clearer understanding of AUD in nurses include the following:

- Alcohol addiction falls under the umbrella of SUD, rather than as a separate (and, thus, quantifiable) disease, which can be diagnosed using the DSM-5 criteria.^5^,^9^,^10^
- Underreporting (nurses do not admit they have a problem, privately self-treat, or are fearful to report).^9^
- Social acceptability of alcohol use.
- Underdiagnosis in healthcare settings by physicians.^11^,^12^

## Underdiagnosed and undertreated

The National Institutes of Health (NIH) defines AUD as “a medical condition characterized by an impaired ability to stop or control alcohol use despite adverse social, occupational or health consequences.”^13^ Yet despite the incidence of AUD and its inclusion in the DSM-5, it remains underdiagnosed and undertreated in the general population.^5^,^11^,^12^ In fact, most people who have the disorder do not receive treatment for it, even when they disclose their drinking problem to their primary care provider or another HCP.^12^

Increased alcohol consumption has been normalized in the medical field. A recent nationwide study by the Washington University School of Medicine found that physicians do not often treat addiction issues in their patients with AUD. The researchers found that in their sample, about 80% of people with AUD had utilized healthcare for a variety of reasons in the previous year, and roughly 70% of them were subsequently screened about their alcohol use. However, only a minority received care: 11.6% reported receiving a brief intervention, roughly 5% were referred to treatment, and roughly 6% received treatment.^11^

Another study found that primary care physicians underdiagnose patients with alcohol abuse as well as underregister them in medical records.^12^ The study reported that of the 179 males and 82 females who met the criteria for AUD via patient-reported data, only 1 of the males and none of the females were documented as such by the primary care physician. The authors hypothesized that this was due to barriers related to alcohol screening and the stigma around alcohol abuse.^12^

In addition, most physicians fail to use effective medications for the treatment of AUD. In a 2021 NIH study, researchers found that only 1.6% of the millions of Americans with AUD had been prescribed a medication to help them control their drinking.^14^ The authors concluded that despite the effectiveness of the medications for addiction treatment, physicians rarely prescribe them, in part because many physicians are not trained in addiction or educated on the approved medications for treatment.^14^

It may have been unclear how physicians and other HCPs should address the issue of alcohol addiction because there were two diagnoses in the DSM-IV: alcohol abuse and alcohol dependence. However, in 2013, the DSM-5 simplified the disease classification into one diagnosis. AUD is classified by severity (mild, moderate, or severe) through 11 criteria that address drinking habits and consequences (see *DSM-5 AUD diagnostic criteria*).^5^,^15^

## COVID-19 and AUD

Nurses are increasingly asked to work in challenging environments that place them in positions vulnerable to stress and psychological trauma.^3^ In March 2020, when it became clear that SARS-CoV-2 had spread to the US, the resulting stress of the COVID-19 pandemic on frontline caregivers created the perfect storm of vulnerability for addictive behaviors.^3^

The burden of the pandemic atop an already stress-laden hospital environment only worsened preexisting compassion fatigue, burnout, and the feeling of being simultaneously overworked and undervalued.^16^ Many of these factors including understaffing, feeling inadequate at the job, lack of support, and workplace bullying have been linked to alcohol use in nurses for coping with professional stress.^3^

Delivering patient care during the pandemic has been especially difficult due to the convergence of high patient census; inadequate staffing; shortages of both personal protective equipment and mechanical ventilators; lack of clinical guidelines and protocols; feelings of isolation; and the anger, despair, and sometimes verbal abuse directed at HCPs.

In the worst scenarios, HCPs were called upon to decide who received care. This contributed to a phenomenon referred to as moral injury, which is a feeling of having betrayed or violated one's own personal or professional morals and beliefs by acting in a certain way due a set of circumstances that are out of the person's control. In the healthcare setting, this refers to not being able to provide optimal patient care.^16^ Moral injury and other COVID-19-related factors create a perfect storm for the development and relapse of AUD.^17^

## Barriers to treatment

All 50 states have ethical and legal regulations regarding substance and alcohol use among nurses. This includes specific requirements for reporting to the state BON, disciplinary or nondisciplinary actions, alternative-to-discipline (ATD) processes, and monitoring for the transition back to work.^9^,^18^,^19^

ATD programs offer the opportunity for nurses and HCPs to pursue treatment and monitoring programs that theoretically avoid the punitive measures they would face from traditional measures, such as termination.^3^ These programs also have high recovery and return-to-practice rates, especially after 5 years in the program with ongoing drug monitoring and various support group meetings.^20^ However, barriers like termination persist and keep a large portion of nurses with AUD from seeking treatment. Thus, despite the success of these programs, national enrollment rates among nurses remain staggeringly low, according to Marvin Seppala, MD, a national expert in addiction treatment. (Video communication with permission, December 10, 2021.)

Seppala identified several key barriers to nurses seeking treatment:

- Failure of physicians to recognize AUD, partly due to a false perception that AUD is not a disease and a lack of training to recognize and assess signs and symptoms.
- Fear of reporting suspected alcohol abuse among colleagues.
- Monetary losses, including job loss (getting fired because of addiction); cost of residential (inpatient) and outpatient treatment programs; and the mandatory long-term outpatient services that often include 2-5 years of drug testing, psychotherapy, and other services.
- Stigma around the disease, which physicians, nurses, and other HCPs consider a character flaw rather than a treatable disease.
- Loss of healthcare coverage, which can be due to job loss.
- Loopholes that allow payers to deny coverage for AUD treatment programs and services, despite the existence of the Affordable Care Act and the Mental Health Parity and Addiction Equity Act, which offer SUD/AUD coverage like that of other diseases.^21^
- Lack of positive messaging about the effectiveness of ATD programs.

Seppala also pointed out that the systems in place generally protect physician but not nurse employment. Physicians stayed longer at the Hazelden Betty Ford Foundation, an addiction treatment center, than nurses because of the former's ability to cover the costs. This was also partly due to many nurses being fired rather than being allowed to continue their job. (Marvin Seppala, MD, video communication with permission, December 10, 2021.)

## Finding solutions

A common-sense start would be to implement the recommendations of the American Nurses Association's official position statement, which calls upon healthcare facilities to educate nurses and other staff on substance and alcohol abuse; to cultivate safe, supportive, and drug-free workplaces via solid policies and practices; and to adopt ATD approaches to treating nurses and nursing students with SUD and AUD.^22^ Goals should include retention, rehabilitation, and reentry into safe, professional practice. Nurses and nursing students should have a thorough understanding of substance abuse risks, drug diversion, and their responsibility and means to report suspected or actual substance abuse.^22^

Tactical approaches to these initiatives include:

- **A national public relations (PR) campaign to reduce AUD stigma**. Because stigma is a primary barrier to treatment, a PR campaign led by the medical and nursing communities and other national stakeholders could transform the landscape of addiction treatment for society at large. An example is a 2021 campaign called *Life Unites Us*, piloted in Pennsylvania for combating stigma related to opioid use disorder (OUD). It significantly improved attitudes toward medication-assisted treatment and overall views of individuals struggling with OUD.^23^ Widespread understanding that addiction is not a moral failure or a choice, but rather a progressive disease that is preventable and treatable, is essential to change the trajectory of this disease.^24^ Stigma-awareness campaigns could play a role in significantly reducing stigma and increasing access to treatment.
- **Conduct scientific studies on nurses with AUD**. Using a diagnostic tool specific to AUD will enable the collection of concrete data, providing the foundation for strong and supportive policies and procedures, and a receptive climate to the diagnosis, treatment, and recovery of nurses with AUD.
- **Offer workplace educational programs**. Ensure that every hospital across the US has sophisticated employee health programs for mental health and addiction, including AUD.^25^ A variety of educational resources on SUD for nurses and nurse managers available through the National Council of State Boards of Nursing (NCSBN) could be adapted for AUD.^18^ Furthermore, develop a collaborative, multidisciplinary approach across the healthcare system so education, programs, and support are readily accessible.
- **Implement compassionate, shame-free national ATD policies that protect nurses**. Policies that protect nurses' licenses and their jobs while they get help need to be written, supported, and implemented nationally. Having a national, standardized ATD program pathway for nurses can help all involved.^20^ Reducing the stigma can help pave the path for nurses to come forward about their addictions. Nurses who went through ATD programs can help by advocating for these programs and sharing helpful experiences (see *Nonprofit AUD recovery resources*).
- **Allocate public or private funding so nurses can afford treatment**. This can include special monetary compensation for treatment for nurses who worked during the pandemic.
- **Have support staff dedicated to healthcare workers' psychological needs**. The heightened vulnerability of nurses during the SARS-CoV-2 pandemic requires careful clinical attention to mental health issues and easy access to counseling and psychotherapy.^6^ This includes hiring addiction specialists, counselors, and spiritual advisors working within hospitals to support the nursing staff. Some hospitals created a *Code Lavender:* an evidence-based relaxation and restoration intervention system used when challenging situations threaten unit stability, personal emotional equilibrium, or professional functioning.^26^
- **Bolster collegiate education for nurses and physicians**. Collegiate curricula need to reflect the current frequency, severity, and treatment of AUD and SUD. For example, the University of Minnesota's College of Nursing offers a module on addiction, SUD, and AUD as part of their required element of the program's final “Nurse in Transition to Practice” curriculum.^27^ Students will learn to identify the signs and symptoms of SUD and how to recognize behaviors in themselves and their colleagues. Physicians should be alerted to recognize potential AUD by using the DSM-5 criteria.

With these types of initiatives in place, nurses like Sarah would be better positioned to get the help they need in a timely and efficient manner.

In Sarah's case, it took about 10 years to reinstate her nursing license. The process included legal fees and required arduous rounds of approvals and proof of attendance in recovery group meetings even after 2 years of sobriety.

## Conclusion

Sarah's story has a happy ending, professionally, but it also includes chapters on prison time and loss of marriage, income, family, and housing in addition to her involuntary career hiatus. Sarah's case study reveals the compelling need to convert an outdated shame-and-blame model into a treatable-disease model that has substantial support, treatment, and clarity of process.

Nurses have a golden opportunity to lead the charge in helping colleagues with AUD through their leadership, personal experiences, clear communications, and collaboration with all stakeholders. Immediate action on these initiatives could advance education about AUD as well as the identification and compassionate treatment of AUD among nurses.

## Interview questions on the history of AUD and workplace support

1. Briefly describe the onset of your AUD including contributing factors.
2. Did the conditions of your job contribute to your AUD progression? If so, explain.
3. Did AUD ever affect your work such as absenteeism or being intoxicated while working? If so, explain.
4. What led you to seek treatment, and was your employer involved in any way?
5. What have been your experiences and observations with alternative-to-discipline (ATD) programs either personally or through colleagues?
6. How has your employer or profession supported you either getting treatment, during treatment, or while waiting to be reinstated after treatment, such as in employee health programs, Nurses Peer Support Network, and supervisors?
7. Do you have suggestions or solutions to improve the process for nurses who need help with AUD?
8. Do you feel alcohol use has increased, decreased, or remained the same among colleagues?
9. How does your profession perceive others with a SUD?

## DSM-5 alcohol use disorder (AUD) diagnostic criteria

**The 11 symptoms of AUD**

A problematic pattern of alcohol use leading to clinically significant impairment or distress, as manifested by at least two of the following, occurring within a 12-month period:

1. Alcohol is often taken in larger amounts or over a longer period than was intended.
2. There is a persistent desire or unsuccessful efforts to cut down or control alcohol use.
3. A great deal of time is spent in activities necessary to obtain alcohol, use alcohol, or recover from its effects.
4. Craving, or a strong desire or urge to use alcohol.
5. Recurrent alcohol use resulting in a failure to fulfill major role obligations at work, school, or home.
6. Continued alcohol use despite having persistent or recurrent social or interpersonal problems caused or exacerbated by the effects of alcohol.
7. Important social, occupational, or recreational activities are given up or reduced because of alcohol use.
8. Recurrent alcohol use in situations in which it is physically hazardous.
9. Alcohol use is continued despite knowledge of having a persistent or recurrent physical or psychological problem that is likely to have been caused or exacerbated by alcohol.
10. Tolerance, as defined by either of the following: a) A need for markedly increased amounts of alcohol to achieve intoxication or desired effect; b) A markedly diminished effect with continued use of the same amount of alcohol.
11. Withdrawal, as manifested by either of the following: a) The characteristic withdrawal syndrome for alcohol; b) Alcohol (or a closely related substance, such as a benzodiazepine) is taken to relieve or avoid withdrawal symptoms.

**AUD is classified by severity based on the number of symptoms**.

**Mild:** The presence of 2 to 3 symptoms.

**Moderate:** The presence of 4 to 5 symptoms.

**Severe:** The presence of 6 or more symptoms.

Reprinted with permission from the *Diagnostic and Statistical Manual of Mental Disorders, Fifth Edition*, (Copyright © 2013). American Psychiatric Association. All Rights Reserved.

**Table:** AUD treatment and recovery resources

*Alternative-to-Discipline Programs* **National Council of State Boards of Nursing (NCSBN)** www.ncsbn.org/alternative-to-discipline.htm NCSBN hosts an online database of ATD programs for SUD. ATD programs (state-based) for substance/AUD enhance a BON's ability to quickly assure public protection. The benefits to the nurse include the opportunity to demonstrate to the BON in a nondisciplinary and nonpublic manner that they can become safe and sober and remain so while retaining their license.
*Nonprofit Treatment Programs* **Cornerstone of Recovery** www.cornerstoneofrecovery.com/alcoholhism-drug-addiction-treatment-program-nurses At Cornerstone of Recovery, the Professionals Program is designed specifically for nurses. This entails medical detoxification and a general treatment program. **Caron: Healthcare Professionals Program** www.caron.org/treatment-programs/healthcare-professionals HCPs designed the program for the specific needs and concerns of all HCPs including nurses. **Hazelden Betty Ford** www.hazeldenbettyford.org/education/bcr/addiction-research/health-care-professionals-substance-abuse-ru-615 The Hazelden Betty Ford Foundation offers comprehensive addiction treatment programs designed specifically for HCPs, including a first-of-its-kind specialized program for nurses.
*Nurses Peer Support Networks* In the US, 43 states have a Nurses Peer Support Network, which can be affiliated with the state BON or the state nurses association or be completely independent. In these volunteer organizations, nurses in recovery support other nurses in active addiction or early sobriety. *Nonprofit Recovery Support*
**She Recovers Foundation** https://sherecovers.org; www.facebook.com/groups/SHERECOVERSSupportHealthcare (a support group for healthcare professionals) She Recovers aims to end the stigma and shame often associated with recovery to promote healing and growth. It connects women through its virtual platforms and in-person community networks and supports them in developing holistic recovery patchworks. **Alcoholics Anonymous (AA)** www.aa.org AA is an international fellowship of individuals who experienced drinking problems. Its program is based on the 12 steps, a group of principles, spiritual in their nature, which, if practiced as a way of life, can expel the obsession to drink and support an alcohol-free life. Note: There are many other 12-step programs for other addictive substances and behaviors. **Self-Management and Recovery Training (SMART) Recovery** www.smartrecovery.org This international nonprofit assists individuals seeking abstinence from addiction. It uses the SMART approach, a secular and research-based strategy that employs cognitive behavioral therapy and nonconfrontational motivational methods. **Refuge Recovery** www.refugerecovery.org Inspired by the guiding philosophy for the program are the teachings of Buddhism, Refuge Recovery is a practice, a process, a set of tools, a treatment, and a path to healing from addiction.

This is not an endorsement of a particular program or organization, but rather a compilation based on information in the public domain.
